# Enhancing Therapeutic Efficacy against *Brucella canis* Infection in a Murine Model Using Rifampicin-Loaded PLGA Nanoparticles

**DOI:** 10.1021/acsomega.3c07892

**Published:** 2023-12-13

**Authors:** Karol Yesenia Hernández-Giottonini, Beatriz Arellano-Reynoso, Rosalva Josefina Rodríguez-Córdova, Jonathan de la Vega-Olivas, Efrén Díaz-Aparicio, Armando Lucero-Acuña

**Affiliations:** † Posgrado en Nanotecnología, Departamento de Física, Universidad de Sonora, Hermosillo 83000, Mexico; ‡ Departamento de Ingeniería Química y Metalurgia, Universidad de Sonora, Hermosillo 83000, Mexico; ⊥ Facultad de Medicina Veterinaria y Zootecnia, Universidad Nacional Autónoma de México, Circuito Exterior Ciudad Universitaria, Coyoacán, Ciudad de México 04510, Mexico; ○ CENID Salud Animal e Inocuidad, Instituto Nacional de Investigaciones Forestales, Agrícolas y Pecuarias, Carretera Federal México-Toluca Km. 15.5, Cuajimalpa, Ciudad de México 05110, Mexico

## Abstract

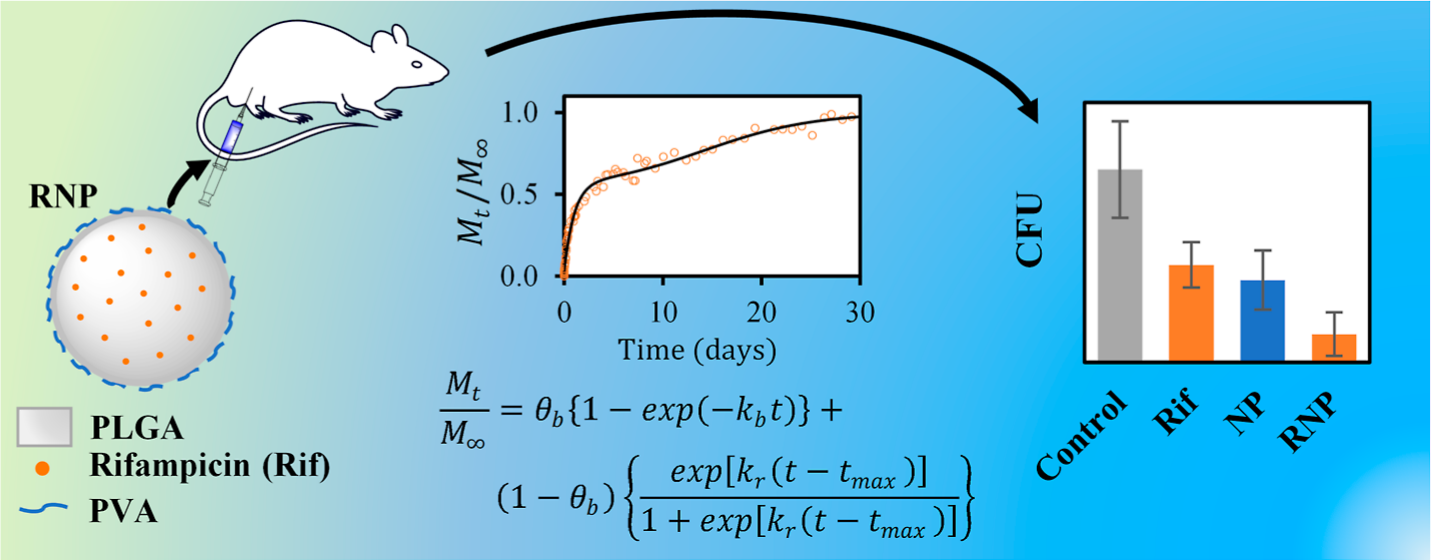

The *in vivo* efficacy of rifampicin encapsulated in poly(lactic-*co*-glycolic acid) (PLGA) nanoparticles was evaluated for the treatment of BALB/c mice experimentally infected with *Brucella canis*. The PLGA nanoparticles loaded with rifampicin (RNP) were prepared using the single emulsification-solvent evaporation technique, resulting in nanoparticles with a hydrodynamic diameter of 138 ± 6 nm. The zeta potential and polydispersity index values indicated that the system was relatively stable with a narrow size distribution. The release of rifampicin from the nanoparticles was studied in phosphate buffer at pH 7.4 and 37 °C. The release profile showed an initial burst phase, followed by a slower release stage attributed to nanoparticle degradation and relaxation, which continued for approximately 30 days until complete drug release. A combined model of rifampicin release, accounting for both the initial burst and the degradation–relaxation of the nanoparticles, effectively described the experimental data. The efficacy of RNP was studied *in vivo*; infected mice were treated with free rifampicin at concentrations of 2 mg per kilogram of mice per day (C1) and 4 mg per kilogram of mice per day (C2), as well as equivalent doses of RNP. Administration of four doses of the nanoparticles significantly reduced the *B. canis* load in the spleen of infected BALB/c mice. RNP demonstrated superior effectiveness compared to the free drug in the spleen, achieving reductions of 85.4 and 49.4%, respectively, when using C1 and 93.3 and 61.8%, respectively, when using C2. These results highlight the improved efficacy of the antibiotic when delivered through nanoparticles in experimentally infected mice. Therefore, the RNP holds promise as a potential alternative for the treatment of *B. canis*.

## Introduction

Infectious diseases have consistently remained a significant global threat to public health over the past few decades.^1^ Among the various infectious diseases, brucellosis stands out as a widespread infectious zoonosis that is observed worldwide. Recognized by the World Health Organization as one of the most severe bacterial diseases transmitted between animals and humans, brucellosis continues to pose a serious threat to public health around the world, particularly in developing nations.^2−4^ Brucellosis has a significant impact on animal and human health in endemic regions, with *Brucella abortus* (*B. abortus*), *Brucella melitensis*, *Brucella suis*, and *Brucella canis* being the main causative agents of this infection in humans.^5,6^ Human cases of *B. canis* typically result from exposure to reproductive tissues and fluids, accidental laboratory infections, or contact with infected dogs.^7^ Moreover, *B. canis* is recognized as the primary cause of reproductive failures in dogs, leading to substantial economic losses in breeding kennels due to abortions, stillbirths, and sperm abnormalities.^8,9^*Brucella* species are characterized as facultative intracellular pathogens that can invade the cells of the mononuclear phagocyte system, evade intracellular killing mechanisms, and establish their replicative niche before the activation of adaptive immunity.^10^ Consequently, treating brucellosis presents a challenge for physicians as it requires prolonged therapy with a combination of antimicrobial drugs.^11^

Given the limitations of conventional treatments for brucellosis, alternative approaches have been explored, including the use of a drug delivery system based on nanoparticles. Nanoparticles have demonstrated notable enhancements in terms of permeability, targeted drug delivery efficiency, decreased toxicity, and other favorable characteristics when employed as drug delivery systems (DDS).^12,13^ Poly(lactic-*co*-glycolic acid) (PLGA) is a biodegradable and biocompatible copolymer approved by the United States Food and Drug Administration (US FDA) for use in several DDS, for biomedical use.^14,15^ The PLGA nanoparticles show potential as DDS for a wide range of therapeutic agents toward different ends (antiseptics, antibiotics, and anti-inflammatory and antioxidant drugs).^16^ Several studies have investigated the encapsulation of specific drugs in nanoparticles as potential treatments for brucellosis.^17−19^ One such drug under investigation is rifampicin, which acts on bacterial polymerase by forming a stable drug–enzyme complex that inhibits bacterial DNA transcription.^20^ However, the use of rifampicin is limited due to several drawbacks, including its low bioavailability, which demands the administration of large doses throughout treatment. This prolonged administration can lead to significant side effects, such as skin, liver, and hypersensitivity reactions.^21,22^

In this study, we prepared rifampicin-loaded PLGA nanoparticles (RNP) using a single emulsification-solvent evaporation technique. Nanoparticles were characterized by dynamic light scattering, laser Doppler electrophoresis, and scanning electron microscopy. The *in vitro* drug release behavior was determined under physiological conditions. The release profile was analyzed using a biphasic release model, considering an initial burst phase and degradation–relaxation of nanoparticles. This release model was employed to predict the doses of nanoparticles used in the *in vivo* study. We tested the efficacy of the nanoparticles and the free drug in BALB/c mice experimentally infected with *B. canis* RM6/66, measuring colony-forming units (cfu) and spleen weight as indicators. Our study demonstrates that rifampicin-loaded PLGA nanoparticles have the potential to enhance the efficacy of the free drug against *B. canis*.

## Materials

PLGA with a molecular weight of 17 kg mol^–1^ and an inherent viscosity of 0.16–0.24 dL/g was obtained from Corbion Purac, Gorinchem, The Netherlands. Poly(vinyl alcohol) (86–89% hydrolysis, low molecular weight, PVA) was obtained from Alfa Aesar, Ward Hill, Massachusetts, USA. Phosphate buffered saline (PBS) tablets were obtained from Sigma-Aldrich, St. Louis Missouri, USA. Dichloromethane (DCM) was purchased from Fisher Scientific Inc., Fair Lawn, New Jersey, USA. Rifampicin, >98%, *M*_w_ of 822.95 g mol^–1^ was purchased from TCI, Tokyo, Japan.

### Preparation of Rifampicin—PLGA Nanoparticles

PLGA nanoparticles (NP) and rifampicin—PLGA nanoparticles (RNP) were prepared by the single emulsification-solvent evaporation technique.^23,24^ Briefly, 50 mg of PLGA and 2.5 mg of rifampicin were dissolved in an organic solution (DCM). Next, 25 mL of an aqueous solution of 3% PVA was added to the organic phase. The mixture was emulsified for 1 min at 75% amplitude (90 μm) under an ice bath by using a QSonica 500 ultrasonicator (QSonica LLC, Newtown, Connecticut, USA). The organic solvent was evaporated under magnetic stirring at room temperature. Then, the solution was washed by three centrifugation cycles using a Sigma 3-30KS centrifuge (Sigma Laborzentrifugen GmbH Osterode am Harz, Germany) operated at 37,565*g* (20,000 rpm) for 20 min. On the final centrifugation cycle, the nanoparticles were resuspended in deionized water, characterized, and freeze-dried in a lyophilizer FreeZone 2.5 L Benchtop (Labconco, Kansas City, Missouri, USA) using sucrose as a cryoprotectant.

### Characterization of Nanoparticles

The size, polydispersity index (PDI), and zeta potential (ζ) of NP and RNP were evaluated at 25 °C using a Zetasizer Nano ZS equipment from Malvern Instruments, Ltd. (Worcestershire, UK). Dynamic light scattering (DLS) was employed for measuring the size of the nanoparticles with a refractive index of 1.33 and water serving as the dispersant. Each sample underwent three measurements with each measurement consisting of 10 runs. Laser Doppler electrophoresis was utilized to measure the ζ. The size average and ζ values were obtained from three independent experiments. The surface morphology of the nanoparticles was examined through field emission scanning electron microscopy (SEM) using a Hitachi S-4800 FE-SEM instrument (Hitachi Corporation, Tokyo, Japan). To prepare the samples, a small quantity of lyophilized nanoparticles was placed on a double-sided carbon tape affixed to an SEM stub. Loose nanoparticles were removed by using compressed air. A platinum coating was applied by using an Anatech Hummer 6.2 sputter system (Anatech USA, Hayward, California, USA). The process involved 60 s under 10 mA of argon plasma. Nanoparticles were visualized using a beam strength of 1.0 kV and a working distance ranging from 8 to 9 mm.

### *In Vitro* Rifampicin Release

The *in vitro* release profile of rifampicin from RNP was determined by using UV–vis spectroscopic analysis. The release study was conducted under sink conditions using a dialysis membrane method. Before use, the membrane was soaked in deionized water for 20 min. Freeze-dried RNP were dispersed in 1 mL of 10 mM sodium phosphate buffer (pH 7.4) containing 1% DMSO using a bath sonicator. The RNP dispersion was then placed in a dialysis bag with a molecular weight cutoff of 12,000–14,000 Da (Spectrum Laboratories Inc., Rancho Dominguez, CA, USA). The dialysis bag was incubated at 37 °C in 30 mL of PBS containing 1% DMSO. At predetermined time intervals, 1 mL of the incubation medium was withdrawn and immediately replaced with the same volume of PBS with 1% DMSO. The withdrawn samples were analyzed for rifampicin content using spectrophotometry at 337 nm on a UV–vis Genesis 10S instrument (Thermo Scientific, Madison, Wisconsin, USA). The experiment was performed in triplicate. The mass of rifampicin encapsulated into the RNP, known as the drug loading (DL), and the encapsulation efficiency (EE) were determined from the drug release data using eqs 1 and 2, respectively. The total mass of rifampicin released from the RNP was considered the final mass of the drug encapsulated into the nanoparticles.
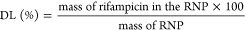
1
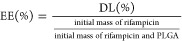
2

### Drug Release Analysis

The release of rifampicin from the RNP was analyzed in an experimental study, focusing on a biodegradable system. Several factors were considered to understand the drug-release behavior of these nanoparticles.^25^ The release profile of drugs from PLGA nanoparticles typically exhibits multiple phases to encompass the different mechanisms involved in the release process.^26^ These mechanisms include the initial burst and degradation of the polymer, among others. The initial burst mechanism involves interfacial diffusion between the nanoparticle surface and the surrounding liquid medium. Various factors can influence this process, such as drug–drug and polymer–drug interactions, as well as interphase properties.^27^ However, the solubility and concentration of the drug near the surface of the nanoparticles play a crucial role. In the case of rifampicin, it has been reported to have limited aqueous solubility,^28^ suggesting that only a small amount of the drug will be released in a short period.

The initial burst phenomenon can be understood by considering that the rate of drug release is proportional to the amount of dissolved drug with all of the effects combined into a proportionality constant known as the initial burst constant (*k*_b_). Assuming that initially, all the rifampicin was encapsulated within the nanoparticles, the kinetics of the initial burst follow an exponential relationship.^26,29^
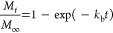
3where *M*_*t*_ is the cumulative mass of rifampicin released at time *t* and *M*_∞_ is the cumulative mass of rifampicin released at infinite time. The mechanism of drug release by the degradation of the PLGA particles could have a dependency on the pH,^30^ the temperature,^31^ the size of the PLGA nanoparticles,^32^ among other factors. In literature, the degradation–relaxation of PLGA nanoparticles by adapting the empirical Prout–Tompkins equation has been explained,^33,34^ which can be written as follows
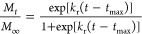
4where *k*_r_ is the rate of degradation–relaxation constant and *t*_max_ is the time to achieve a maximum rate of rifampicin release or the time to achieve 50% of release. Thus, the experimental release of rifampicin could be analyzed with a linear combination of eqs 3 and 4, considering a biphasic and simultaneous drug release, as presented in eq 5.
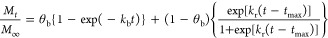
5where the initial burst contribution fraction over the entire rifampicin release process is θ_b_ and the fraction of degradation–relaxation θ_r_ is the remaining release contribution or (1 – θ_b_). The unknown parameters of eq 5, θ_b_, *k*_b_, *k*_r_, and *t*_max_ are determined by adjusting the equation to the experimental release of rifampicin from RNP by a nonlinear least-squares algorithm in MATLAB (R2010b, MathWorks, Natick, MA, USA).

### Preparation of Bacterial Suspension and Infection by *B. canis*

For the experimental infection study, the *B. canis* RM6/66 virulent strain was utilized. The bacterial inoculate was prepared by seeding it in trypticase soy broth (TSB) and incubating it at 37 °C in 5% CO_2_ for 18 h. Subsequently, the bacterial culture was washed with sterile PBS (pH 7.4) and centrifuged at 6000 rpm for 15 min. The supernatant was discarded, and the pellet was resuspended in 10 mL of PBS. Dilutions were then made from the bacterial suspension to obtain the desired infectious dose. Ultimately, a volume of 300 μL, containing a concentration of 1.6 × 10^8^ colony-forming units per milliliter (cfu/mL), was used to infect the mice via intraperitoneal injection.

### Animals and Experimental Design

All animal procedures took place at the “CENID Salud Animal e Inocuidad, Instituto Nacional de Investigaciones Forestales, Agrícolas y Pecuarias”, of Mexico City. These procedures were in accordance with institutional guidelines and with approval from the Institutional Animal Care and Use Committee of the “Universidad Nacional Autónoma de México”, protocol 657. A total of twenty-eight female BALB/c mice, aged between six and 8 weeks, were used as experimental models. The mice were acclimated for at least 1 week before the start of the experiments and were provided with ad libitum access to food and water throughout the study. The experimental design consisted of seven groups, each consisting of four mice (*n* = 4). One group received only PBS with 1% DMSO. The remaining groups were administered free rifampicin, RNP, and NP at two different concentrations. For the free rifampicin treatment, two concentrations of the drug were tested: 2 and 4 mg of rifampicin per kilogram of mice per day. The amount of RNP used for each treatment was calculated based on the weight of the mice and the DL of the nanoparticles. The same amount of NP was administered in each case. In all treatments, the nanoparticles were dispersed in PBS with 1% DMSO. The treatment initiation was set for 14 days after the inoculation and administered via the intraperitoneal route. Four treatment doses were given at 14, 16, 18, and 20 days postinfection. The mice were inoculated with 300 μL of the free rifampicin solution or the corresponding nanoparticle solution. After 11 days from the last treatment, the mice were euthanized, and their spleens were collected in a sterile technique. The spleens were weighed and homogenized with 1 mL of sterile PBS. Serial dilutions were prepared in a microplate and plated on *Brucella* agar. The plates were incubated at 37 °C with 5% CO_2_ for 72 h. After the incubation period, cfu counts were performed for each plate.

### Data Analysis

The efficacy of the treatments was determined by counting the number of cfu per milliliter and the weight of the mice’s spleen. Statistical analysis for the efficacy of the treatments was analyzed by ANOVA one-way analysis of variance to compare the control and treated groups. *P* values of <0.05 were considered statistically significant.

## Results and Discussion

### Characterization of NP and RNP

In this study, NP and RNP were prepared by using the single emulsification solvent evaporation technique. The obtained values for various parameters are summarized in Table 1, including average particle size, PDI, ζ, DL, and EE. The average particle size and PDI were found to be similar for both preparations regardless of the drug used. This finding confirms the reproducibility of the nanoparticle preparation technique employed. Additionally, the PDI values for both preparations were below 0.1, indicating a narrow size distribution that aligns with the therapeutic objectives of this study. However, there were noticeable differences in the zeta potentials among the preparations, although the system remained relatively stable. The mean zeta potential value for RNP was slightly higher than that for NP, suggesting some adsorption of rifampicin on the surface or near the surface of the nanoparticles. The DL was determined to be 2.4 ± 0.8%, meaning that each milligram of RNP contained 0.024 mg of rifampicin. The EE resulted in values of 47.8 ± 16.1%. In a similar study by Vibe et al., rifampicin was encapsulated in PLGA nanoparticles for combating mycobacterial infection in zebrafish, with a drug loading efficiency of 31.8% (drug weight to total nanoparticle weight).^35^ Maghrebi et al. conducted a study in which they reported an encapsulation efficiency of 24.2 ± 0.1% for rifampicin-loaded PLGA nanoparticles.^36^ Overall, the characterization results indicate successful preparation of the nanoparticles with consistent particle sizes, narrow size distributions, and reasonable drug loading and encapsulation efficiencies. These findings are in accordance with previous studies on rifampicin-loaded nanoparticles and demonstrate the potential of the developed formulation for various therapeutic applications.

**Table 1:** Characteristics of the NP and RNP Formulations (Means ± SD, *n* = 9)^a^

sample	size (*d*, nm)	PDI	ζ (mV)	DL (%)	EE (%)
NP	137.5 ± 2.6	0.073 ± 0.020	–24.1 ± 3.2		
RNP	137.5 ± 6.1	0.064 ± 0.020	–20.3 ± 3.8	2.4 ± 0.8	47.8 ± 16.1

a The drug loading and encapsulation efficiency are also shown. (means ± SD, *n* = 3.)

### Surface Morphology

**Figure 1:**
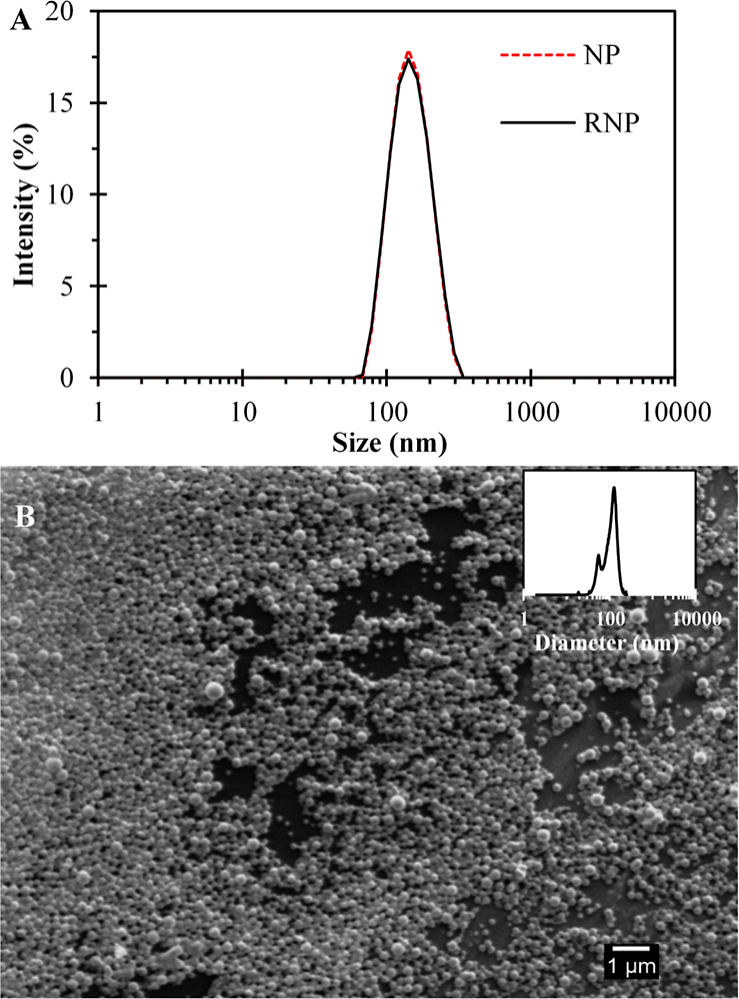
Characterization of the nanoparticles. (A) Dynamic light scattering spectra of NP and RNP were used to determine the average diameter and polydispersity index of each nanoparticle based on an average of ten measurements. (B) Scanning electron micrograph images of a NP.

SEM analysis was conducted to examine the morphological characteristics of dried PLGA nanoparticles prepared by using the single emulsification method. The obtained micrograph, as shown in Figure 1, revealed that the NP exhibited a spherical shape and displayed a uniform size distribution. The surface of the nanoparticles appeared smooth and free from any observable defects, indicating the successful production of high-quality nanoparticles through the preparation process. To further analyze the particle size, a histogram was generated using ImageJ 1.8.0_112 software based on the analysis of over 200 particles. The histogram showed an average diameter of 113 ± 43 nm, which was consistent with the size measured by DLS analysis, where a hydrodynamic diameter of 137.5 ± 2.6 nm was obtained. It is important to note that DLS measures the hydrodynamic diameter in solution, while SEM captures the dried particle morphology. Similar findings have been reported in other studies involving rifampicin-loaded nanoparticles prepared by using the emulsification technique. In these studies, SEM images also demonstrated regular spherical shapes with smooth surfaces, although the specific sizes varied depending on the experimental conditions.^37−39^

### *In Vitro* Rifampicin Release

**Figure 2:**
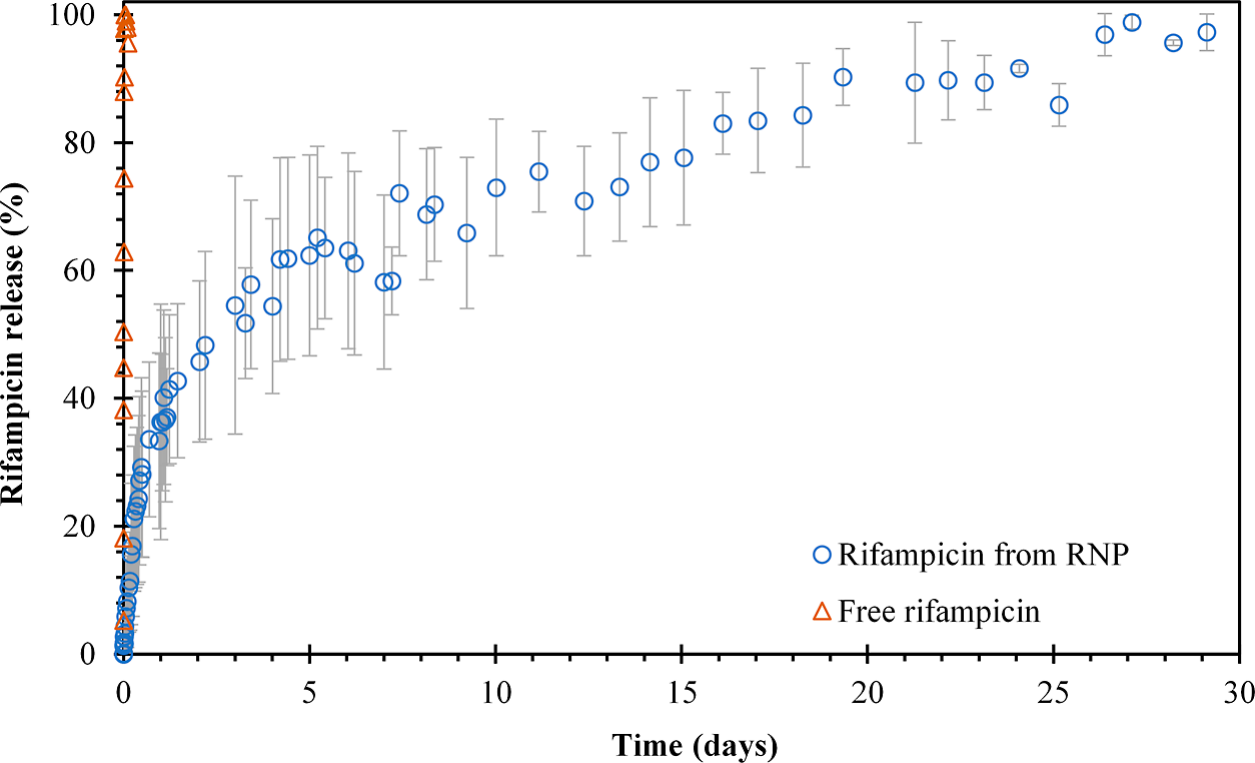
Experimental release of rifampicin from the RNP (blue ○) and the control of free rifampicin release from the dialysis membrane (orange Δ), performed in 10 mM PBS buffer at pH 7.4 and 37 °C. The data represent the mean ± SD (*n* = 3).

The release of rifampicin from RNP was evaluated in PBS with 1% DMSO at pH 7.4 and 37 °C. The release profile of rifampicin from RNP exhibited a biphasic pattern, characterized by an initial burst release in the first 2 days, accounting for approximately 40% of the total drug release (Figure 2). This initial phase of release primarily stems from the drug molecules located near the surface of the nanoparticles and is influenced by the solubility of rifampicin and the concentration gradient between the nanoparticles and the buffer solution. Following the initial burst release, a slower release phase was observed, leading to complete drug release within 30 days of the study. This second phase of release is attributed to the nanoparticle degradation–relaxation and the subsequent increase in porosity, facilitating the diffusion of rifampicin molecules through the polymeric matrix of the RNP. The sustained release observed during this phase is a result of the medium penetrating into the matrix, causing continuous hydrolysis and degradation of the PLGA.^40^ The dialysis membrane permeability to rifampicin was evaluated as a control using a free drug under the same experimental conditions mentioned above. The results demonstrated that all the free drug diffused through the dialysis membrane within the first few hours of the study, as depicted in Figure 2. The focus was primarily on the release behavior of rifampicin from the RNP system rather than on the permeability characteristics of the dialysis membrane. Therefore, the mass transfer effects related to the transport of rifampicin through the dialysis membrane were neglected in this analysis.

### Drug Release Analysis

**Figure 3:**
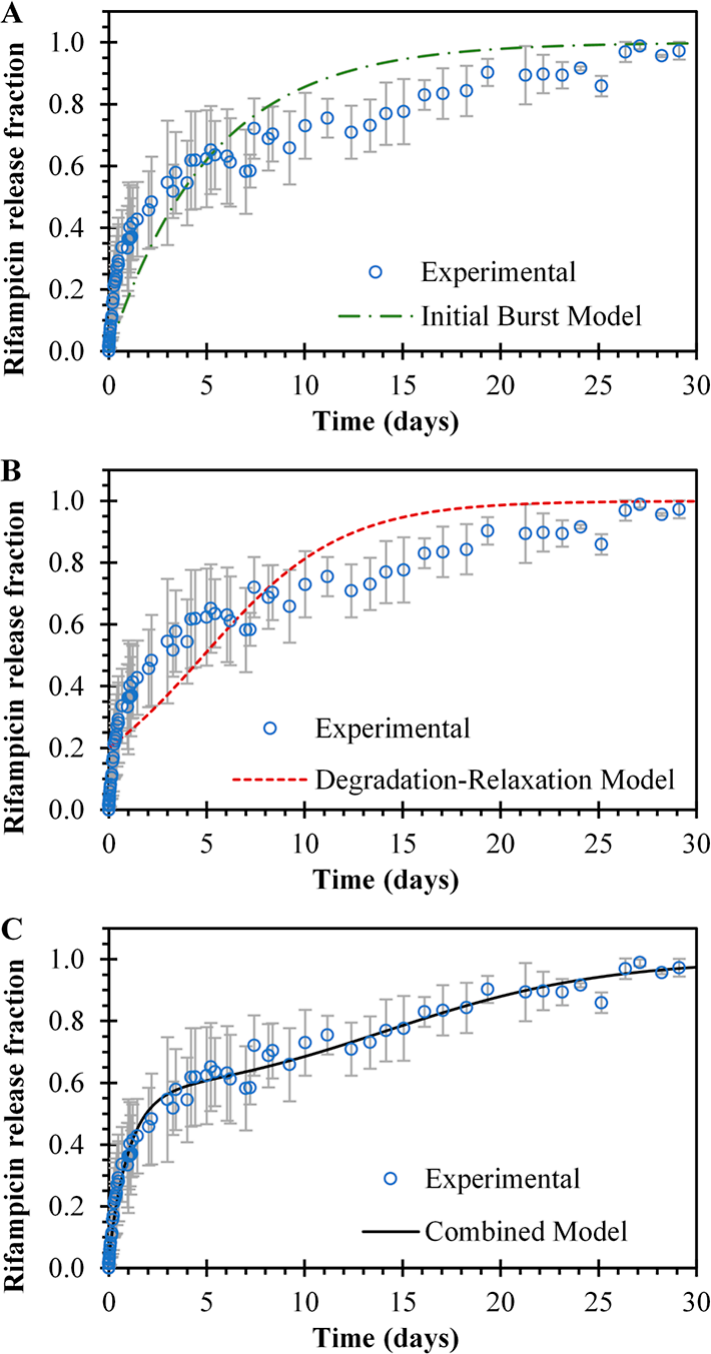
Experimental and theoretical rifampicin release profile from RNP in 10 mM PBS buffer at pH 7.4 and 37 °C. (A) Circles (blue ○) represent experimental data, and the square dot line (green -•-•-•-) represents fitting to the experimental data when the initial burst model of eq 3 was applied. (B) Dashed–dotted line (red ----) represents the degradation–relaxation model of eq 4, and (C) solid line represents fitting to experimental data when the combined model is considered, eq 5.

Figure 3A illustrates the model fitting to the experimental data when only the contribution of the initial burst effect is considered, as indicated in eq 3. The long dashed–dotted line represents this fitting. In Figure 3B, the dotted line represents the fitting of the mathematical model to the experimental data when the release mechanism is solely governed by the nanoparticle degradation–relaxation, as indicated in eq 4. The combined model, shown in Figure 3C, considers the simultaneous effect of the initial burst and nanoparticle degradation–relaxation, analyzed using eq 3. The combined model demonstrates an effective fit to the rifampicin release experimental data, as indicated by the outstanding coefficient of determination, as shown in Table 2. Notably, the burst constant experiences an increase when the combined model is employed in comparison to the initial burst model. Conversely, the degradation–relaxation constant exhibits a decrease when the combined model is utilized as opposed to that of the degradation–relaxation model. In contrast, the time required to achieve a 50% release displays an opposing trend. These adjustments to the parameters of the combined model enable it to better conform to the experimental data, resulting in a slightly greater influence of the initial burst model in comparison with the degradation–relaxation model. The need for multiple simultaneous release mechanisms to explain the release profile of hydrophobic drugs from biodegradable nanoparticles has been previously reported by Lucero-Acuña and Guzmán.^26^

**Table 2:** Parameters of Rifampicin Release Determined and Used in the Mathematical Development of the Model from PLGA Nanoparticles

parameter	description	unit	model		
			initial burst	degradation–relaxation	combined
*k*_b_	burst constant	days^–1^	0.1937		0.9863
θ_b_	fraction of burst release		1		0.5356
*k*_r_	degradation–relaxation constant	days^–1^		0.2843	0.1793
*t*_max_	time to achieve 50% of release	days		4.8360	14.1395
θ_r_	fraction of degradation–relaxation			1	0.4644
*R*^2^	coefficient of determination		0.9247	0.9176	0.9948

### Efficacy of Treatments against *B. canis* Infected Mice Model

**Figure 4:**
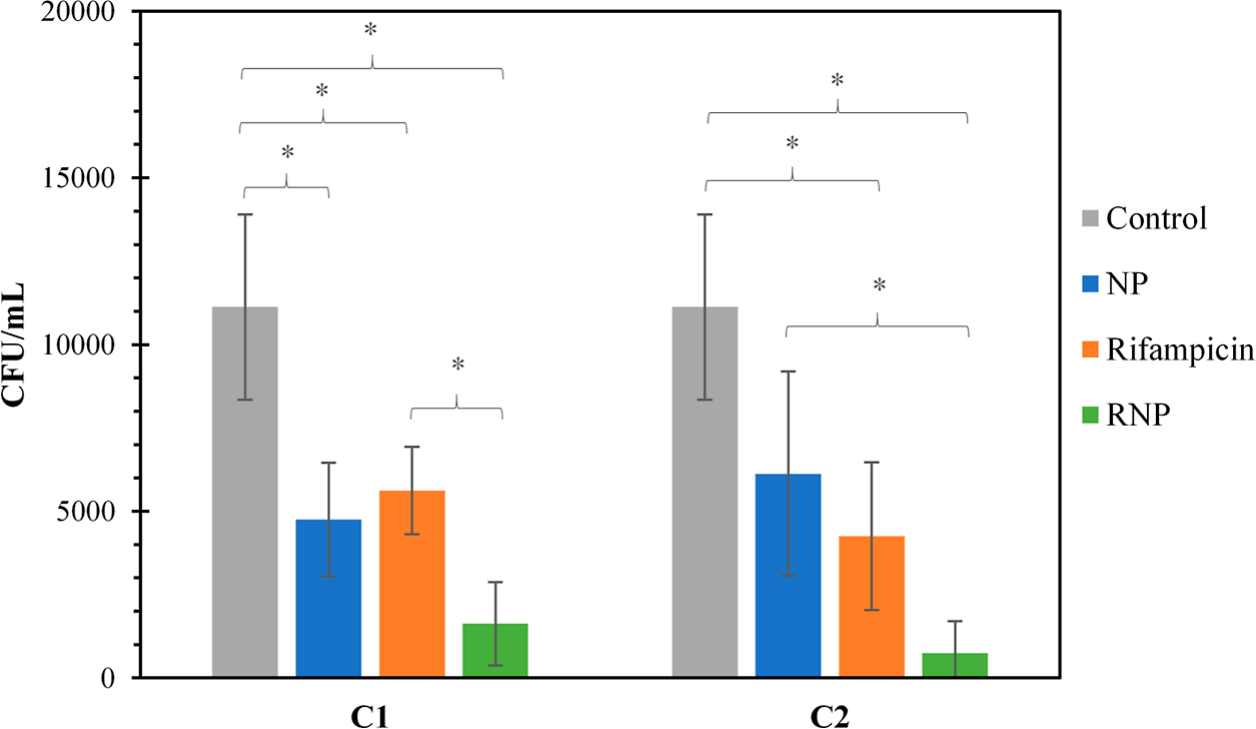
*In vivo* infection assay. Intracellular *B. canis* reduction (cfu/mL) was observed in infected BALB/c mice after four doses of treatments. The data represent the mean ± SD (*n* = 4). C1: concentration of 2 mg of rifampicin/kg of mice/day of treatment and C2: concentration of 4 mg of rifampicin/kg of mice/day of treatment. Treatments: control (gray ■), NP (blue ■), rifampicin (orange ■), and RNP (green ■). (*P < 0.05.)

To determine the efficacy of RNP against *B. canis* infection, infected mice were treated with free rifampicin at concentrations of 2 mg per kilogram of mice per day (C1) and 4 mg per kilogram of mice per day (C2), as well as equivalent doses of NP and RNP. The control group consisted of mice inoculated with PBS and 1% DMSO. The efficacy of each treatment was assessed by measuring the cfu/mL count in the spleens (Figure 4). In the case of C1, all treatments exhibited a statistically significant difference when compared to the control group (*P* < 0.05).

In contrast, for C2, treatments involving free rifampicin and RNP displayed a significant difference, while the NP did not exhibit a significant variance compared to the control group. In the case of NP treatment, a decrease in the cfu/mL count was observed compared to the control group for both concentrations. However, the effect of the nanoparticles was not directly proportional to their concentration. Also, it is important to note that phagocytic cells are responsible for eliminating foreign particles, including nanoparticles, through phagocytosis.^41^ After phagocytosis, nanoparticles can stimulate phagocytic cells and induce host defense reactions.^42−44^ Therefore, the reduction in cfu/mL caused by NP may be attributed to their stimulating effect on the immune system. Additionally, it is worth mentioning that the decrease in cfu count in the spleen could also be influenced by the natural course of the infection. Previous studies have shown that colonization of the spleen gradually decreases in mice infected with *B. canis*, and the bacteria persist for several weeks in a dose-dependent manner.^45^ There was no significant difference between NP C1 and RNP C1, which could be attributed to the low concentration of rifampicin in RNP (concentrations up to 25 mg of rifampicin per kilogram of mice per day are used in the literature for mice).^46^ However, RNP C2 showed a significant reduction compared to NP C2 (*P* = 0.018), supporting the observations. For C2, there was no statistically significant difference found between the treatments using free rifampicin and RNP. However, it is worth mentioning that a notable reduction in bacteria was observed in the spleens of mice that received the RNP treatment. In fact, on some plates, there was a complete absence of cfu counts. On the other hand, both free rifampicin and RNP treatments led to a decrease in cfu/mL counts compared to the control group. Notably, RNP demonstrated superior effectiveness compared to free drugs, achieving reductions of 85.4 and 49.4%, respectively, when using C1 and 93.3 and 61.8%, respectively, when using C2. Furthermore, the mathematical rifampicin release model used in this work indicated that at the time of euthanasia, the total amount of rifampicin had not been completely released from the nanoparticles, reaching only around 80% for the initial dose and lower percentages for the other ones. These results indicate that RNP were more effective than free rifampicin in reducing the number of *B. canis* cfu in the spleen of mice even when compared to a higher amount of the free drug. It highlights that NP and free rifampicin demonstrated a therapeutic effect, but combining these treatments in the form of RNP potentiated the decrease in cfu. The effectiveness of RNP can be attributed to the encapsulation of the drug in the nanoparticles, which allows for sustained release. It is worth mentioning that *Brucella* can efficiently colonize cells of the monocyte/macrophage lineage and replicate in high numbers in the liver and spleen.^47,48^ Phagocytic cells internalize particles more efficiently than other host cells, which results in the accumulation of nanoparticles in the mononuclear phagocyte system (MPS) organs, such as the liver and spleen.^49^ This accumulation becomes advantageous for targeting intracellular infections that affect the MPS, such as brucellosis.^50^ Therefore, nanoparticles not only improve the efficacy of current treatments but also reduce adverse effects and mitigate drug resistance, common issues in this type of infection. Additionally, considering that rifampicin release from the RNP lasted for 30 days in vitro, it is presumed that nanoparticles could reduce the dosing frequency and improve patient compliance. In a similar study, Imbuluzqueta et al. evaluated the efficacy of hydrophobic gentamicin-loaded PLGA nanoparticles against *B. melitensis* infection in mice and found that these systems can sustain therapeutic concentrations of GEN-AOT in the liver and spleen.^51^ On the other hand, Prior et al. observed that gentamicin-loaded PLGA 50:50H microspheres did not exhibit therapeutic activity in mice infected with *B. abortus* 2308, which might be attributed to inappropriate particle size (∼3 μm) and aggregation.^52^

Other researchers have investigated the efficacy of nanoparticle systems *in vitro*. For example, Bodaghabadi et al. demonstrated that rifampicin-loaded nanocarriers increased the efficacy of rifampicin in reducing the number of *B. melitensis*.^53^ Similarly, Ghaderkhani et al. evaluated solid lipid nanoparticles loaded with rifampin and found statistically significant antibacterial activity in bacterial and cell culture media compared to free rifampicin.^54^ Hosseini et al. reported that doxycycline-loaded solid lipid nanoparticles were more effective in reducing the number of *B. melitensis* cfu in macrophages compared to free doxycycline, suggesting that the use of nanoparticles ensures continuous and consistent drug presence at the target site.^55^ Additionally, Seleem et al. compared the efficiency of nanoplexes with free streptomycin and doxycycline using the J774.A1 macrophage-like cell line infected with *B. melitensis*, providing evidence for the ability of nanoplexes to penetrate cell membranes and target intracellular *B. melitensis*.^56^

**Figure 5:**
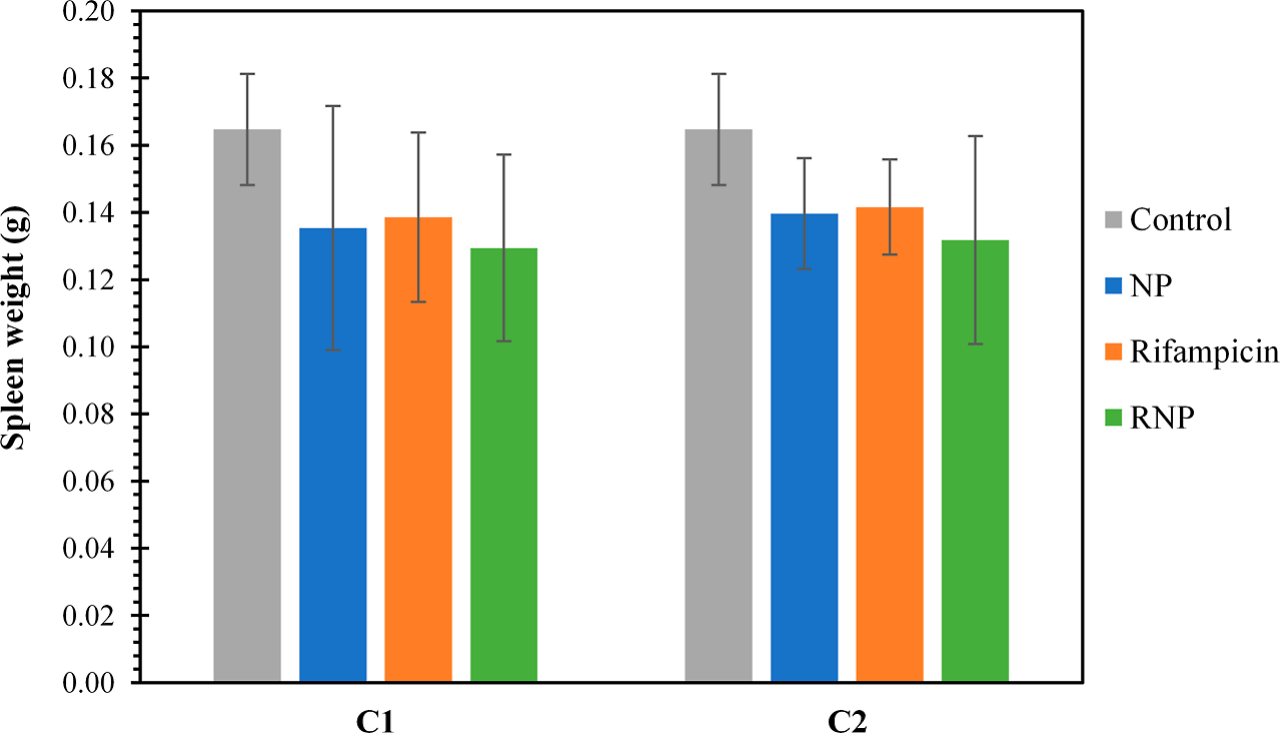
Effect of the treatments in the weight of the spleens of *B. canis*-infected mice. Mice were treated with four doses of treatments. C1: concentration of 2 mg of rifampicin/kg of mice/day of treatment and C2: concentration of 4 mg of rifampicin/kg of mice/day of treatment. Treatments: control (gray ■), NP (blue ■), rifampicin (orange ■), and RNP (green ■). The data represent the mean ± SD (*n* = 4).

After the treatments, the spleens were collected and weighed. The results of the spleen weight measurements are presented in Figure 5. As mentioned earlier, the weight of the spleen is a crucial parameter as splenomegaly, characterized by an enlarged spleen, is a common feature of brucellosis.^57^ No statistically significant differences were observed between the different treatment groups, likely due to the limited number of samples. Nonetheless, a trend of decreased spleen weight can be observed in the mice receiving treatment compared to the control group. Notably, the groups of mice treated with RNP exhibited slightly lower spleen weights, with the most significant effect observed at a concentration of 4 per kilogram of mice per day. These findings support the results obtained from the cfu count, where the RNP demonstrated the highest efficacy in reducing cfu in the spleen. Therefore, it can be suggested that the decrease in the spleen weight in mice infected with *B. canis* is directly linked to the elimination of a larger number of bacteria within it. Consistent with our results, Seleem et al. found that nanoparticles loaded with streptomycin and doxycycline were more effective than free drugs in reducing the *B. melitensis* load in the spleens and livers of infected BALB/c mice.^56^ However, future studies should include a comparison with a noninfected mice group as a benchmark for spleen size. Table 3 presents the spleen weights after treatments and the corresponding reduction effects on the cfu count for each treatment. Also, the statistical differences discussed above between the treatment groups are summarized in Table 4.

**Table 3:** Effect of Four Doses of the Different Treatments against Infection with *B. canis* RM6/66 Administered Intraperitoneally^a^

treatment	C1: 2 mg rifampicin			C2: 4 mg rifampicin		
	spleen wt (g)	cfu/mL	reduction (%)	spleen wt (g)	cfu/mL	reduction (%)
control	0.17 ± 0.02	11,125 ± 2780		0.17 ± 0.02	11,125 ± 2780	
NP	0.16 ± 0.04	4750 ± 1707	57.3	0.14 ± 0.02	6125 ± 3065	44.9
free rifampicin	0.15 ± 0.02	5625 ± 1315	49.4	0.14 ± 0.01	4250 ± 2217	61.8
RNP	0.14 ± 0.02	1625 ± 1250	85.4	0.13 ± 0.03	750 ± 957	93.3

a Results were obtained 11 days after the administration of the last dose. Reduction percentages are concerning to the control.

**Table 4:** *P*-Values in Comparisons between the cfu Results for the Treatment Groups of Each Concentration Analyzed: NP, Rifampicin, RNP, and the Control (*P < 0.05)

comparison	C1 *P* values	C2 *P* values
control-NP	2.01 × 10^–^^3^*	5.15 × 10^–^^2^
control-rifampicin	6.19 × 10^–^^3^*	7.47 × 10^–^^3^*
control-RNP	5.60 × 10^–^^5^*	2.60 × 10^–^^4^*
NP-rifampicin	9.09 × 10^–^^1^	6.92 × 10^–^^1^
NP-RNP	1.37 × 10^–^^1^	3.51 × 10^–^^2^*
rifampicin-RNP	4.50 × 10^–^^2^*	2.19 × 10^–^^1^

## Conclusions

NP and RNP were successfully prepared by using a single emulsification method followed by solvent evaporation. The characterization results affirm the successful preparation of the nanoparticles, showcasing uniform particle sizes, narrow size distributions, and suitable drug loading and encapsulation efficiencies. The release profile of rifampicin from RNP exhibited a distinctive biphasic pattern: an initial burst release within the first 2 days followed by a more gradual release phase, ultimately resulting in a complete drug release over 30 days. This release pattern underscores the promising potential of this studied system for the sustained administration of rifampicin; it is presumed that nanoparticles could reduce the dosing frequency and enhance patient compliance. Furthermore, a combined model incorporating the contributions of the initial burst and the degradation–relaxation of nanoparticles was found to be a good fit to describe the experimental rifampicin release data. This release model was employed to predict the doses of nanoparticles used in the *in vivo* study. Remarkably, an *in vivo* study revealed that the use of PLGA nanoparticles as a carrier for rifampicin led to improved treatment outcomes compared with free rifampicin. This was noteworthy, especially when considering that the mathematical rifampicin release model suggested that, at the time of euthanasia, only around 80% of the initial dose of rifampicin had been released from the nanoparticles, with even lower percentages for subsequent doses. RNP could also reduce adverse effects and mitigate drug resistance. Moreover, a trend of decreased spleen weight was discernible in the mice receiving RNP treatment compared to the control group. These results align with the findings from the cfu count, indicating that RNP was the most effective at reducing cfu in the spleen. These findings highlight the capacity of PLGA nanoparticles to enhance the efficacy of rifampicin against intracellular infections such as *B. canis*.

## Abbreviations

NP: nanoparticles
RNP: rifampicin-loaded PLGA nanoparticles
DL: drug loading
EE: encapsulation efficiency
